# Gangliosides as Therapeutic Targets for Neurodegenerative Diseases

**DOI:** 10.1155/2024/4530255

**Published:** 2024-04-08

**Authors:** Orhan Kerim Inci, Hande Basırlı, Melike Can, Selman Yanbul, Volkan Seyrantepe

**Affiliations:** ^1^Department of Molecular Biology and Genetics, Izmir Institute of Technology, Gulbahce Campus, Urla, 35430 Izmir, Türkiye; ^2^Izmir Institute of Technology, IYTEDEHAM, Gulbahce Campus, Urla, 35430 Izmir, Türkiye

## Abstract

Gangliosides, sialic acid-containing glycosphingolipids, are abundant in cell membranes and primarily involved in controlling cell signaling and cell communication. The altered ganglioside pattern has been demonstrated in several neurodegenerative diseases, characterized during early-onset or infancy, emphasizing the significance of gangliosides in the brain. Enzymes required for the biosynthesis of gangliosides are linked to several devastating neurological disorders, including Alzheimer's disease (AD), Parkinson's disease (PD), Huntington's disease (HD), amyotrophic lateral sclerosis (ALS), hereditary spastic paraplegia (HSP). In this review, we summarized not only the critical roles of biosynthetic enzymes and their inhibitors in ganglioside metabolism but also the efficacy of treatment strategies of ganglioside to address their significance in those diseases.

## 1. Introduction

**Figure 1:**
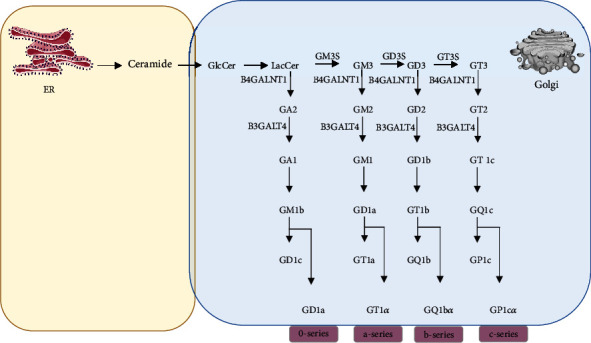
Illustration of the summary of ganglioside biosynthetic pathways in the cellular mechanism. Svennerholm's nomenclature is used for the identification of gangliosides. Gangliosides, containing 0, 1, 2, and 3 sialic acid residues connected to the innermost galactose, are found in the 0, a-, b-, and c-series, respectively. Colored boxes indicate the areas where sphingolipids and gangliosides are produced. GM3S: CMPNeuAc:lactosylceramide alpha-2,3-sialyltransferase; GD3S: ST8alpha-N-acetylneuraminide alpha-2,8-sialyltransferase 1; B4GALNT1: UDP-Gal:betaGlcNAc beta-1,4 N-acetylgalactosaminyltransferase 1; B3GALT4: UDP-Gal:betaGlcNAc beta 1,3-galactosyltransferase 4.

Gangliosides, sialic acid-containing glycosphingolipids (GSL), are primarily found in the outer leaflet of the plasma membrane, particularly in the nervous tissues of vertebrate cells. They are involved in differentiation, cell growth, cell signaling, and cell-cell communication [1]. Gangliosides also play essential roles in neuronal regeneration, memory formation, synaptic transmission, and neurogenesis [2–4]. Gangliosides are synthesized by stepwise addition of monosaccharide units to lactosylceramide precursor, which is produced in the cytoplasmic leaflet of the endoplasmic reticulum and then transported to the Golgi apparatus [5] (Figure 1). Gangliosides are abundant, especially in the nervous tissue. The brain's gangliosides are composed of four complex gangliosides (GM1, GD1a, GD1b, and GT1b) to a greater extent than 90% [6]. Several neurodevelopmental and neurodegenerative problems in both human and mouse models are caused by mutations that result in the loss of function of the enzymes required in ganglioside biosynthesis [7, 8]. Alteration of the specific ganglioside levels (Table 1) and treatment strategies using specific gangliosides (Table 2) were reported in severe neurodegenerative disorders such as Alzheimer's disease, Parkinson's disease, Huntington's disease, amyotrophic lateral sclerosis (ALS), and hereditary spastic paraplegia (HSP) [9–13]. This review summarized the current knowledge of altered ganglioside metabolism related to abnormal neuropathologies in common neurodegenerative disorders.

### 1.1. Alzheimer's Disease

Alzheimer's disease (AD) is the most common neurodegenerative disorder of the central nervous system (CNS) that causes dementia. AD is neuropathologically characterized by neuronal death, senile plaques of aggregated amyloid-*β* (A*β*), and neurofibrillary tangles (NFT) made of hyperphosphorylated tau protein [46]. There is still no effective cure for AD [47]. AD is due to gene mutations in several genes, including APP, presenilin 1 (PSEN1), and presenilin 2 (PSEN2) [48–50]. A bunch of studies demonstrated that alterations in ganglioside metabolism are related to AD pathophysiology in patients and transgenic mouse models [51, 52]. Patients with familial or early-onset AD have considerably lower total ganglioside levels in the brain's frontal white matter and gray matter. On the other hand, only the temporal cortex, hippocampus, and frontal white matter of patients with late-onset or sporadic AD showed reduced total ganglioside levels [14]. Besides, the levels of ganglioside GM1 are lower, while ganglioside GM3 levels are more abundant in the brains of AD patients [53]. Moreover, in some AD patients, the frontal and parietal cortex had a higher level of minor gangliosides like GM2, GM3, GD3, and GM4 [51]. Fukami et al. reported that b-series gangliosides, GT1a, and GQ1b, unique markers for cholinergic neurons, were also present in higher amounts in the brains of AD patients [54]. Lower GD1b and GT1b levels were also noted in the hippocampus gray matter of AD patients [15].

In contrast to AD patients, the alteration of ganglioside metabolism in transgenic AD mouse models remains unclear. Sawamura et al. reported that mutant presenilin-2 mice have no discernible changes in the major brain gangliosides; however, they had a strikingly raised level of A*β*1-42 [55]. APP/PSEN1-double transgenic mice model did not display alteration of a- and b-series of ganglioside levels compared to control mice [16]. APP^SL^ transgenic mice cortex showed higher amounts of GM2 and GM3 ganglioside levels apart from reduced levels of GQ1b, GD1b, GT1a, and GD3 gangliosides in the cortex [52]. The 14-month-old 5xFAD transgenic mice model has significantly increased the GM2, GM3, GT1b, and GD1a levels in the hippocampus and cortex regions [18]. To clarify ganglioside metabolism in AD, Oikawa et al. demonstrated that deletion of the N-acetylgalactosaminyltransferase (*B4galnt1*) gene in the 1xFAD transgenic mice model displayed accumulation of the GM3 and GD3 ganglioside but lacking GM1, GD1a, GD1b, GT1b, and GQ1b gangliosides. Accumulation of the GM3 and GD3 or the absence of GM1 and GD1a exacerbated the disease's neuropathology [9]. Another study showed that 2xFAD (hAPP/PS-1) mice with GD3-Synthase (*St8sia1*) deficiency accumulate plenty of GM1, GD1a, and GM3 gangliosides despite lacking GD1b, GD3, GT1b, and GQ1b. St8sia1-deficient 2xFAD mice demonstrated an improvement in AD pathology in comparison to B4galnt1-deficient 1xFAD animals [16]. Dukhinova et al. generated 5xFAD mice with GM3-synthase (*St3gal5*) deficiency, which did not have major gangliosides (GM1, GD1a, GD3, GT1b, and GQ1b). Like the 2xFAD/St8sia1 double knock-out mice model, 5xFAD/St3gal5 double knock-out mice exhibited lower A*β* deposition and neuroinflammation and no sign of neuronal death [17]. Herzer et al. used 5xFAD//Ugcgf/f//Thy1-CreERT2//EYFP mice, which has the deletion of glucosylceramide synthase (GCS) in forebrain neurons, which improved dendritic spines in the dentate gyrus and elevated memory tasks [18]. In addition, much evidence has been found that the activities of glucosyltransferases affect AD neuropathology. Overexpression of the B4galnt1 causes the elevation of ganglioside levels and induces APP processing by blocking BACE1 degradation in lysosomes [56]. In addition to the modeling that targets ganglioside metabolism, research has also been conducted using various molecules to treat AD in vitro and in vivo. The reduction of A*β* deposition is targeted by inhibiting GM1 ganglioside indirectly using leptin, which ameliorated AD pathology in detergent-resistant membrane microdomains (DRMs) of neurons through the phosphatidylinositol 3-kinase/Akt/mammalian target of rapamycin pathway [57]. Few studies have been conducted in vitro and in vivo to determine the utility of GSL synthesis inhibitors targeting gangliosides as antiamyloidogenic agents. First, the synthetic ceramide analog D-1-phenyl 2-decanoylamino-3-morpholino-1-propanol (D-PDMP) inhibitor significantly reduced A*β* secretion from SHSY5Y neuroblastoma cells [58]. Another study showed that other related ceramide analog GSL inhibitors based on the PDMP structure reduced the secretion of human APP695 (CHO-APP) expressed from CHO cells and A*β* from primary human neurons [59, 60]. In contrast, Takasugi et al. showed that D-PDMP upregulated A*β* production from HEK293 cells stably expressing the Swedish mutant of APP and Neuro2a cells, respectively, whereas N-butyldeoxynojirimycin (NB-DNJ) did not [61]. In an in vivo study, Wang et al. confirmed that elevated GM1 ganglioside levels cause amyloid plaque accumulation and cognitive dysfunctions. Based on this result, different strategies were tested to reduce GM1 ganglioside levels, especially in APP/PS1 mice. It has been shown that using the glycosphingolipid inhibitors D-PDMP and CTB to prevent ganglioside accumulation in APP/PS1 mice significantly reduced A*β* levels in APP/PS1 mice [35]. In addition, GENZ 667161, which is a GCS (glucosylceramide synthase) inhibitor, was administered with a diet that reduced soluble A*β*-42 and amyloid plaque burden [37]. Apart from these, the effects of sialic acids on A*β* accumulation have been examined. For this purpose, intracranial injection of Neu1 sialidase into the 5XFAD mouse model of AD was found to reduce the number of A*β* plaques and A*β* peptide levels. Therefore, Neu1 sialidase was identified as a risk factor for developing AD-like amyloidosis [62]. In addition, Neu3 sialidase overexpression was also found to aggravate cognitive impairment in APP/PS1 mice due to increased GM1 ganglioside levels [35]. Moreover, sialic acid-specific lectin of *Limax flavus* agglutinin injection to the 5XFAD mice improved cognitive test performance, amyloid depositions, and neuroinflammation [17]. The therapeutic potential of direct injection of gangliosides has been applied in many studies in AD. Intramuscular injection of GM1 ganglioside for 24 weeks and 6 weeks did not improve disease symptoms and cognitive functions in AD patients, respectively [28, 29]. Svennerholm displayed that intrathecal injection of GM1 ganglioside for 12 months halted disease pathology for AD patients [30]. Moreover, intracerebroventricular injection of GM1 for the patients mitigated physical activities and cognitive functions [31, 32]. Intraperitoneal administration of GM1 ganglioside to the APP/PS1 mouse model exhibited a reduction of the A*β*-40 and A*β*-42 [33]; however, a recent study showed that intraperitoneal GM1 administration caused pathological abnormalities in APP/PS1 mice and failed to rescue cognitive decline [36]. In the Tg2576 mouse model, a monoclonal antibody that specific ganglioside-bounded A*β* was injected through intraperitoneal reduced A*β*-40 and A*β*-42 deposits [34]. In another study, GQ1b administration to the hippocampus of 3xTg-AD mice was shown to reduce APP accumulation and tau phosphorylation, which were associated with decreased APP protein and increased phosphoGSK3*β* levels, respectively [38].

### 1.2. Parkinson's Disease

Parkinson's disease (PD) is a common devastating neurodegenerative disease in which Lewy body formations occur due to *α*-synuclein (*α*Syn) deposition, resulting in the death of dopaminergic neurons, particularly in substantia nigra (SN) [63–65]. PD is caused by genetic and environmental factors and is characterized by disturbed motor functions, including slow movements, impaired gait and balance, bradykinesia, and resting tremors [66]. The SNCA gene encodes presynaptic *α*Syn protein and regulates synaptic functions, neurotransmitter release, and neuroplasticity [67]. Decreased dopamine levels in the brains of patients are a pathological hallmark of PD [68]. *α*Syn-lacking mouse model has reduced dopamine release in the striatum. On the contrary, overexpressed *α*Syn in transgenic mice caused a decrease in the release of dopamine [69, 70].

Various studies have revealed that the binding of *α*-synuclein to the negatively charged lipids through its N-terminal acetyl groups regulates its alpha-helical folding [71]. Studies on inhibiting fibrillar *α*Syn formation due to lipid binding have found a strong relationship between GM1 ganglioside and *α*Syn [72]. Homozygous *B4galnt1*-deficient mice, whose gene is required for the biosynthesis of complex gangliosides such as GM1, showed an abolished level of *α*Syn deposition in the neurons of the SNs [26, 73]. In addition, mice with monoallelic mutations in the *B4galnt1* gene (*B4galnt1^+/-^*) also showed PD-like motor dysfunctions, neurological lesions, and *α*Syn accumulation in the gastrointestinal and cardiovascular systems, similar to PD patients. Studies conducted in PD patient brains determined a significant decrease in the GM1 level of dopaminergic neurons in the SN [11, 21]. Along with measuring the amount of GM1 in the occipital cortex of PD patients by HPTLC, it was determined that there was a significantly less GM1 amount in this region as well [19]. In a study using the colon and heart tissues of PD patients and age-matched controls, significantly reduced levels of GM1 and GD1a were reported. Depleted levels of GM1 and GD1a gangliosides were also detected in skin fibroblast cells of PD patients [20]. In addition to the altered GM1 ganglioside levels in PD patients, the lower expression levels of the ganglioside biosynthetic enzymes, including GM1 synthase (*B3GALT4*) and GD1a/GT1b synthase (*ST3GAL2*), were demonstrated in substantia nigra of PD patients [11, 21].

In a recent study, Akkhawattanangkul et al. demonstrated the effect of reduced proapoptotic GD3 ganglioside in PD mice model induced by 1-methyl-4-phenyl-1,2,3,6-tetrahydropyridine (MPTP). In *St8sia1-/-* mice with the complete absence of GD3 ganglioside, the administration of MPTP did not cause PD pathophysiology [74].

In PD patients, 5-25% of the patients had mutations in the *GBA* gene that encodes lysosomal acid *β*-glucosidase enzyme, and a total of 251 lipids have been analyzed using liquid chromatography/electrospray ionization-tandem mass spectrometry in four brain regions (cingulate gyrus, caudate nucleus, inferior and middle temporal gyrus, globus pallidus). Surprisingly, only significantly altered amounts of gangliosides have been reported [75].

In addition to their implicated roles in the pathology of Parkinson's disease, gangliosides are also used in the potential treatment of PD. In homozygous B4galnt1-deficient mice (*B4galnt1-/-*) which show *α*Syn accumulation in tissues, administration of LIGA-20 is an analog of GM1 that crosses the blood-brain barrier and permeabilizing membrane and has been shown to reduce the *α*Syn accumulation [11]. However, LIGA-20 administration to heterozygous B4galnt1-deficient mice (*B4galnt1+/-*) resulted in reversed cell death of TH^+^ neurons. It reduced *α*Syn accumulation in the SN region, as well as alleviation of PD-like symptoms, particularly movement impairment, in these mice [11]. In another study with rats overexpressing human mutant A53T *α*Syn, GM1 administered to these mice protected against striatal dopamine depletion and dopaminergic neuron death in the SN, reduced *α*Syn accumulation, and improved behavioral abnormalities associated with PD pathophysiology [39]. It was discovered that its hydrophilic oligosaccharide portion exerts the neurotrophic and neuroprotective effects of GM1 ganglioside by interacting with the Trk signaling pathway, named GM1-OS or OligoGM1 [76]. As a result of systemic administration of GM1-OS to heterozygous B4galnt1-deficient mice (B4galnt-1+/-), it was determined that PD-induced physical symptoms were relieved, and decreased *α*Syn level in the SN and tyrosine hydroxylase level increased in these mice [40]. In addition, GM1-OS has been reported to inhibit prion-like and spontaneous *α*Syn accumulation, induce neuronal survival, and protect against *α*Syn accumulation-induced impaired neurite networks in dopaminergic neurons by reducing microglia activation [77]. In a clinical trial with 61 PD patients, 31 patients were given dopasizide as a control group, and 30 patients were administered GM1 ganglioside, which was named “ganglioside” in the study, combined with pramipexole which is an agonist of dopamine receptor [41]. As a result of the study, it was observed that the serum levels of inflammatory markers CRP and TNF-*α* decreased and elevated neurological and motor functions in the patients in the treated group compared to the control group [41]. Intranasal infusion of GM1 into A53T *α*-synuclein-expressing mouse model of Parkinson's disease induced neurogenesis in the adult mice brains, and intranasal infusion of GD3 also promoted the self-renewal ability of neural stem cells [42].

### 1.3. Huntington's Disease

Huntington's disease (HD) is a severe neurodegenerative disease caused by autosomal dominantly inherited mutations in the HTT gene. HD is characterized by progressive motor, cognitive, and psychiatric symptoms [78]. The mutation in the HTT gene leads to the expansion of polyglutamine stretch (polyQ) in the N-terminal of the Huntington protein [10]. Due to mutations in the HTT gene, mutant HTT protein (mHTT) aggregates and causes transcriptional dysregulation, neuronal death, deficits in synaptic activity, and axonal transport [78]. The altered levels of gangliosides were detected in the brains of HD patients [79]. Previous research also indicated reduced ganglioside biosynthetic enzyme activity in the brain of HD mouse models, YAC128 and R6/1 [10, 23]. Furthermore, fibroblast samples from HD patients showed downregulation of ganglioside synthesis enzymes [10]. In the skin fibroblasts of HD patients and the brain of mouse models, the levels of GM1 have been noticeably decreased. It was also found that the levels of GD1a and GT1b gangliosides were reduced in the YAC128 model [10]. Although decreased GM1 levels were detected in caudate nucleus samples of HD patients [23], an increased level of GM1 was demonstrated in the other group of HD patients [22]. The reduced expression levels of *ST8SIA3* which demonstrates homology with GD3-synthase and *B4GALNT1* were detected in the brains of HD patients and the R6/1 mouse model. Additionally, the expression of *ST3GAL5*, which encodes GM3-synthase, and *ST3GAL2*, which encodes GM1b/GD1a/GT1b synthase, was reduced in the caudate of HD patients but not significantly different in the striatum of the R6/1 mouse model [23]. These studies revealed that ganglioside metabolism impairments are associated with HD pathology, and restoration of glycosphingolipid may be a therapeutic approach for HD. Administration of GM1 restored ganglioside levels in HD cells and induced phosphorylation of mutant HTT protein, which resulted in reduced mutant HTT toxicity and improved survival of HD cells [10]. In the YAC128 model, the intraventricular administration of GM1 ganglioside reduced the toxic effects of mutant HTT protein and recovered motor function in mice that were previously symptomatic [43]. Alpaugh et al. showed that intraventricular infusion of GM1 ganglioside ameliorated motor defects, brain atrophy, neurodegeneration, and huntingtin levels in three different HD mouse models: R6/2, Q140, and YAC128 [44]. These studies indicated that chronic intraventricular administration of exogenous GM1 resulted in the recovery of ganglioside levels in the HD mice model and, hence, improvements in motor and cognitive symptoms, normalized levels of neurotransmitters, and diminishment of neurodegeneration [43, 44]. The therapeutic efficacy of GM1 administration could also be explained by its direct effect on mHTT. Exogenous GM1 significantly reduced the levels of both aggregated and soluble forms of mHTT in HT mouse brains without any effect on transcription levels of HTT. Therefore, GM1 ganglioside might be suggested to promote the removal of mHTT at cellular levels [44]. Furthermore, the impact of sphingomyelin (SM) and GM1 ganglioside contents on the interaction between the Huntington protein and lipid membranes has been shown previously. Exon 1 mHTT membrane insertion and the formation of mHTT oligomers on membranes are significantly reduced by the presence of GM1 in artificial membranes created with total brain lipid extract [80].

### 1.4. Amyotrophic Lateral Sclerosis

Amyotrophic lateral sclerosis (ALS) is a lethal neurodegenerative disorder caused by selective degeneration of motor neurons in the motor cortex, brain stem, and spinal cord. The disease causes muscle fatigue, swallowing and speech difficulties, fasciculation, and alterations in reflexes. Individuals with ALS die 3 to 5 years following the onset of the initial disease signs, primarily because of respiratory paralysis. 90% of reported ALS cases have no identifiable cause and are referred to as idiopathic. The other 10% are inherited ALS types caused mainly by autosomal dominant mutations in specific genes [81]. Recently, there has been a focus on the role of GSLs in the progression of ALS since abnormal alterations in GSL homeostasis may contribute to disease etiology [82]. The presence of unique gangliosides [82], high titer serum autoantibodies against GM2 and GM1 [83, 84], and higher levels of GM2 ganglioside are reported in the motor cortex of ALS patients [24]. Dodge et al. also revealed the elevation levels of globotriaosylceramide, ceramide, lactosylceramide, glucosylceramide, galactocerebroside, and the gangliosides GM3 and GM1, as well as the hexosaminidase (HEX) activity in the spinal cords of ALS patients [13]. SOD1^G93A^ mice, a familial model of ALS, also displayed elevated levels of ceramide, glucosylceramide, GM3, and HEX activity [13, 85]. In the study, they demonstrated that, while increasing HEX activity using adenoviral vector administration to the CNS had no impact, the intracerebroventricular injection of GM3 ganglioside dramatically slowed the development of paralysis and prolonged the life of SOD1^G93A^ mice. These results imply that the buildup of GM1 and GM3 gangliosides could have protective effects and might be exploited to halt the course of ALS [13, 85]. Besides, the SOD1^G86R^ mice, a familial model of ALS, exhibit elevated levels of GM1a, GM2, and GM3 gangliosides and phosphatidylinositol but reduced levels of ceramide and glucosylceramide in muscles and spinal cord of the mice model [25, 85]. Inhibition of enzymes of ganglioside catabolism including glucosylceramide beta 2 (GBA2) [86] and *β*-glucocerebrosidase (GCase) [87] in SOD1^G86R^ mice revealed alleviation of disease progression and extension of mouse lifespan. Remarkably, intraperitoneal injection of recombinant natural human IgM (rHIgM12) immunoglobulins targeting GD1a and GT1b prolonged the survival and slowed neurological impairments in two different ALS mice models as SOD1^G86R^ and SOD1^G93A^ in a single dose [45]. Since GD1a and GT1b gangliosides are ligands for myelin-associated glycoprotein (MAG) which is an inhibitor of nerve regeneration, administration of rHIgM12 targeting GD1a and GT1b could prevent MAG-induced suppression of axonal development and repair, thereby enabling neurons to regenerate in mice models of ALS [45]. Therefore, ALS is related to aberrant lipid metabolism [88], and gangliosides and ceramides have been suggested to be disease modulators [83, 84].

### 1.5. Hereditary Spastic Paraplegia

Hereditary spastic paraplegia (HSP) is a group of neurodegenerative diseases that leads to progressive spasticity and weakness of the lower limbs [84]. HSP is classified clinically into pure and complicated forms [89]. The pure form of HSP is characterized by bilateral limb spasticity, impaired vibratory sensation, hyperreflexia, and bladder dysfunction [90]. In addition, cognitive impairments, cerebellar ataxia, neuropathy, and seizures are the clinical signs of complicated forms of HSP. Currently, 79 alleles and genetic loci are known that lead to this disease [91]. The inheritance might be autosomal dominant, autosomal recessive, X-linked, or mitochondrial due to locus heterogeneity [92].

The complex form of HSP (HSP26) is caused by loss-of-function mutations in the *B4GALNT1* gene encoding N-acetylgalactosaminyltransferase [7]. *B4GALNT1* is responsible for synthesizing GM2, GD2, and GA2 by transferring the GalNac unit to the galactose of GM3, GD3, and LacCer. The elevated level of GM3 ganglioside is caused by the deficiency of the B4GALNT1 that cannot compensate for the lack of complex gangliosides. Previously, 10 families with 29 cases were reported as a complicated form of HSP resulting from mutations in the *B4GALNT1* gene [93]. Even though every family has different mutations in the *B4GALNT1* gene, all have a common impairment in complex ganglioside synthesis. The severity of the disease is correlated with the B4GALNT1 activity. The mutations that partially retain the functionality of B4GALNT1 lead to milder symptoms in patients. *B4galnt1*-deficient mice have a deficiency in hippocampal plasticity [27], demyelination, and axonal degeneration, which results in motor and sensory problems [26]. *B4galnt1*-deficient mice also showed neurological symptoms similar to HSP patients. The increased levels of GM3 and GD3 gangliosides were detected in the brain of *B4galnt1*-deficient mice to compensate for the absence of complex gangliosides [12]. Furthermore, the low serum testosterone level and infertility observed in some male patients were also demonstrated in *B4galnt1*-deficient mice [94]. These results suggest that *B4galnt1*-deficient mice could be a suitable model to study the pathogenesis of HSP [95].

## 2. Conclusion

In summary, findings from both human and mouse research clearly show that disruption of biosynthetic enzymes of the gangliosides performs a double-edged effect on the etiology of Parkinson's disease, Alzheimer's disease, Huntington's disease, and hereditary spastic paraplegia. In AD mouse models, the elimination of *St8sia1* and *St3gal5* genes demonstrated enthusing results to reduce the neuropathology of the disease. In the PD mouse model, no MPTP-induced Parkinson's disease neuropathology was observed when *St8sia3* was knocked out. Regarding the other neurological disease, amyotrophic lateral sclerosis, covered in this review, the evidence for the biosynthetic enzymes of gangliosides playing a significant role in etiology is less robust. Finally, the roles of gangliosides concerning neurodegenerative disorders are still unclear, and more research is needed to extend the current state of knowledge.

## Figures and Tables

**Table 1:** Alteration of the ganglioside levels which affect the neuropathology of the neurodegenerative disorders.

Disorder	Species	Model	Ganglioside alterations	Clinical signs	References
AD	Human	Early-onset AD	Total ganglioside	Lower ganglioside level in frontal white and gray matter.	[14]
AD	Human	Late-onset AD	Total ganglioside	Lower ganglioside level in temporal cortex, hippocampus, and frontal white matter of patients.	[14]
AD	Human	AD	GD1b, GT1b	Reduction ganglioside in hippocampal gray matter.	[15]
AD	Mouse	1XFAD/GM2S^−/−^	GM3, GD3	Exacerbation of Alzheimer's pathology	[9]
AD	Mouse	2XFAD/GD3S^−/−^	GM1, GD1a, GM3	Amelioration of disease pathology	[16]
AD	Mouse	5XFAD/GM3S^−/−^	Lack of major gangliosides	Lower A*β* deposition and neuroinflammation	[17]
AD	Mouse	5xFAD//Ugcgf/f//Thy1-CreERT2//EYFP	GM3, GM2, GT1b	Improve memory and loss of dendritic spines	[18]
PD	Human	PD	GM1	Reduction of GM1 in occipital cortex	[19]
PD	Human	PD	GM1, GD1a	Reduction of gangliosides in heart, colon, and skin tissues.	[20]
PD	Mouse	B4GALNT1^−/−^	GM3, GD3	Accumulation of *α*Syn deposition in SN.	[21]
PD	Mouse	B4GALNT1^+/-^	GM1, GD1a, GD1b	PD-like motor functions and *α*Syn accumulation	[11]
HD	Human	HD	GM1	Reduction of GM1 in fibroblast.	[10]
HD	Human	HD	GM1	Increased GM1 in the cerebellum.	[22]
HD	Human	HD	ST8Sia3 and B4Galnt1	Decreased expression of ganglioside synthesis enzymes in the brain.	[23]
HD	Mouse	R6/1	GM1	Molecular, behavioral, and motor disturbances	[22]
HD	Mouse	YAC128	GM1, GD1a, GT1b	Neurodegeneration and motor function abnormalities	[10]
ALS	Human	ALS	GM2	Increased GM2 in the motor cortex	[24]
ALS	Human	ALS	GM3 and GM1	Increased GM3 and GM1 in spinal cord.	[13]
ALS	Mouse	SOD1^G93A^	GM3	Gait impairment and reduced motor function	[13]
ALS	Mouse	SOD1^G86R^	GM1a, GM2, GM3	Spinal motor neuron degeneration, progressive skeletal muscle weakness	[25]
HSP	Mouse	B4GALNT1^−/−^	GM3, GD3	Deficiency of hippocampal plasticity, axonal degeneration	[26, 27]

**Table 2:** In vivo ganglioside-based treatment of neurodegenerative disorders which affect the neuropathology of these disorders.

Disorder	Species	Ganglioside	Route	Clinical signs	References
AD	Human	GM1 ➔ 24 weeks	Intramuscular	No overall symptomatic benefit	[28]
AD	Human	GM1 ➔ 6 weeks	Intramuscular	No improvement in cognitive function.	[29]
AD	Human	GM1 ➔ 12 months	Intrathecal	Disease pathology is halted by continuous treatment.	[30]
AD	Human	GM1	Intracerebroventricular	Improvement of physical activities	[31, 32]
AD	Mouse (APP/PS1)	GM1	Intraperitoneal	Reduction of A*β*-40 and A*β*-42.	[33]
AD	Mouse (Tg2576)	4396C	Intraperitoneal	Reduction of A*β*-40 and A*β*-42 deposits.	[34]
AD	Mouse (APP/PS1)	GM1	Intraperitoneal	Exacerbating cognitive dysfunction	[35, 36]
AD	Mouse (APP/PS1)	AAV-NEU3 (overexpression)	Intracerebroventricular	Accumulation of the A*β* deposits.	[35]
AD	Mouse (APP/PS1)	D-PDMP	Intraperitoneal	Reduction of A*β* deposits and rescue memory.	[35]
AD	Mouse (Tg2576)	GCSi (GENZ 667161)	Diet	Mitigation of the soluble A*β*-42 and amyloid plaque burden.	[37]
AD	Mouse (3xTg-AD)	GQ1b	Intrahippocampal	Reduction of A*β* plaque deposition and tau phosphorylation.	[38]
PD	Mouse (B4galnt1+/-)	LIGA-20	Intraperitoneal	Reduced TH^+^ neuron cell death and *α*Syn accumulation	[11]
PD	Mouse (B4galnt1-/-)	LIGA-20	Intraperitoneal	Reduced *α*Syn accumulation	[21]
PD	Rat (overexpressing human mutant A53T *α*Syn)	GM1	Intraperitoneal	Protection against striatal dopamine depletion and dopaminergic neuron death	[39]
PD	Mouse (B4galnt1+/-)	OligoGM1 (GM1-OS)	Intraperitoneal	Reduced *α*Syn accumulation in SN, increased tyrosine hydroxylase	[40]
PD	Human	GM1 (combined with pramipexole)	Intravenous	Decreased inflammatory CRP and TNF-*α* levels, elevated motor functions	[41]
PD	Mouse (overexpressing human mutant A53T *α*Syn)	GM1 and GD3	Intranasal	Induced neurogenesis and promoted self-renewal ability of neural stem cells	[42]
HD	Mouse (R6/2, Q140, YAC128)	GM1	Intraventricular	Reduced motor defects, neurodegeneration, and huntingtin levels	[43] [44]
ALS	Mouse SOD1^G86R^ and SOD1^G93A^	IgM (rHIgM12) targeting GD1a and GT1b	Intraperitoneal	Prolonged survival and slowed neurological impairments	[45]

## Data Availability

All data used to support the findings of this study are available from the corresponding author upon request.
